# Length of Stay in Mental Health Acute Inpatient Units in Australia vs England: Exploring Differences in Clinical Practice and Service Design

**DOI:** 10.1192/bjo.2025.10493

**Published:** 2025-06-20

**Authors:** Freddie Johansson, James Dove, Eugene Tan, Fran Maffia, Barbara Pavlou

**Affiliations:** 1 North London Foundation Trust, London, United Kingdom; 2 South West Healthcare, Warrnambool, Australia; 3 Royal Free London NHS Foundation Trust, London, United Kingdom

## Abstract

**Aims:** To explore factors in service design that can account for difference in length of stay (LoS) in acute inpatient care for general adult patients between a public mental health service in London, North London Foundation Trust (NLFT) with one in regional Australia, South West Healthcare (SWH).

**Methods:** Information was gathered from the mental health organisations as below:

1. Data comparison of the 2 services over the period Nov 2023 to Dec 2024 relating to patient flow.

2. Comparison of service design in the two systems such as staffing levels, availability of supporting services and clinical practice.

3. Audit in each service comparing factors that can affect LoS.

**Results:** SWH had a shorter length of stay compared with NLFT (13 vs 43 days) in keeping with national and statewide comparison of LoS. Re-admission rates were also lower in SWH (9% vs 15%). There was a significant difference in the number of very long stayers (>60 days) with no such patients in the Australian service.

NLFT had a higher proportion of patients admitted formally (88% vs 65%) and a higher proportion of patients with a psychotic disorder (85% vs 75%).

The service comparison demonstrated higher levels of senior medical input available in the Australian service (1.2 vs 0.6 FTE per 10 patients) and medical staffing in general and more frequent reviews with a Consultant Psychiatrist (4 times weekly in SWH vs once a week in NLFT).

The audit showed more frequent use of high-dose antipsychotic prescribing at discharge (25% vs 18%) and higher amounts of antipsychotic doses in general in the Australian service (79% vs 59% of BNF Maximum Antipsychotic dose) at discharge.

**Conclusion:** The difference in LoS between the services is consistent with benchmarking data. The service evaluation identified several factors that might explain the difference.

There were more patients admitted with psychosis and a higher use of formal admissions in the UK service, both associated with longer LoS.

There were higher levels of medical staffing and in particular Consultant and Registrar levels in SWH. This is likely to explain the difference in frequency of senior reviews for patients in SWH which may result in frequent changes in management plans. The results suggest the use of higher doses of antipsychotic prescribing in SWH.

Staffing models and prescribing practice is likely to impact LoS. It would be important to consider differences in patient experience in the two systems in future evaluations of services.

